# Carbon dioxide elimination as a guide to venoarterial extracorporeal membrane oxygenation weaning: a prospective observational study

**DOI:** 10.1186/s13613-025-01583-4

**Published:** 2025-10-14

**Authors:** Yazhu Yang, Liangshan Wang, Chenglong Li, Hong Wang, Xin Hao, Yan Wang, Yiwen Wang, Ruike Lu, Xiaotong Hou, Zhongtao Du

**Affiliations:** Center for Cardiac Intensive Care, Beijing Anzhen Hospital, Capital Medical University, Beijing, People’s Republic of China

**Keywords:** Cardiogenic shock, Extracorporeal membrane oxygenation, End-tidal carbon dioxide

## Abstract

**Background:**

Despite the increasing use of venoarterial extracorporeal membrane oxygenation (VA-ECMO) in cardiogenic shock (CS), reliable predictors of successful weaning remain poorly defined. This study investigated the role of carbon dioxide elimination parameters in VA-ECMO weaning.

**Methods and results:**

To assess the potential role of end-tidal carbon dioxide (EtCO_2_) in predicting successful VA-ECMO weaning, we conducted a prospective observational study at Anzhen Hospital between January 2023 and December 2024. The primary endpoint was the predictive performance of EtCO_2_ and VCO_2_NL ratio for successful VA-ECMO weaning. Real-time EtCO_2_ monitoring was performed using infrared capnography in mechanically ventilated patients, and the ratio of native lung carbon dioxide elimination (VCO_2_NL) to total carbon dioxide elimination (VCO_2_TOT) was calculated as the VCO_2_NL ratio. Dynamic changes in these parameters were analysed in relation to weaning outcomes. Among 294 patients receiving VA-ECMO for refractory CS during the study period, 91 were included, yielding 562 data points. Both EtCO_2_ (odds ratio [OR] = 1.26, 95% confidence interval [CI] 1.17–1.37, *p* < 0.0001) and the VCO_2_NL ratio (1.14, 95% CI 1.09–1.21, *p* < 0.0001) showed significant correlations with successful weaning. A VCO_2_NL ratio > 79% and EtCO_2_ > 34 mmHg showed strong predictive value for successful weaning (area under the receiver operating characteristics (ROC) curve values of 0.85 [95% CI 0.80–0.89] and 0.84 [95% CI 0.79–0.89], *p* < 0.0001).

**Conclusions:**

EtCO_2_ and the VCO_2_NL ratio may be valuable indicators for predicting successful VA-ECMO weaning. Higher EtCO_2_ and VCO_2_NL ratio values are associated with a greater likelihood of successful weaning.

**Supplementary Information:**

The online version contains supplementary material available at 10.1186/s13613-025-01583-4.

## Background

Venoarterial extracorporeal membrane oxygenation (VA-ECMO) provides vital haemodynamic support for refractory cardiogenic shock (CS) or cardiac arrest (CA), yet optimal weaning timing remains clinically challenging [1–3]. Current weaning decisions balance risks of prolonged support (haemolysis, ischaemia, thromboembolism) against premature withdrawal consequences [1, 4, 5]. Therefore, it is essential to investigate optimal strategy and timing for ECMO weaning, with particular emphasis on monitoring cardiac functional recovery [6, 7].

While echocardiography, pulmonary artery catheters (PAC) and pulse indicator continuous cardiac output (PICCO) monitoring are routinely used, each has significant limitations during ECMO support. Echocardiography offers direct cardiac visualisation but lacks continuous monitoring capability. PAC is invasive and often unreliable in ECMO patients, while PICCO's thermal dilution method may be compromised by ECMO circuit interference [8, 9]. These limitations highlight the need for more robust, real-time cardiac function assessment.

Emerging evidence suggests that the clearance of carbon dioxide (CO_2_) from the native lung during VA-ECMO may serve as a reference indicator for evaluating pulmonary artery blood flow, which correlates strongly with cardiac output [10]. Therefore, in the absence of significant pulmonary disease with stable CO_2_ production, CO_2_ clearance remains stable, and variation in end-tidal carbon dioxide (EtCO_2_) reflects changes in cardiac output [11]. Under VA-ECMO support, the VCO_2_NL ratio (native lung carbon dioxide elimination/total carbon dioxide elimination) (VCO_2_NL/ VCO_2_TOT) may serve as a reliable surrogate for the proportion of native cardiac output relative to systemic perfusion [12]. Continuous monitoring of these parameters may provide real-time insights into cardiac recovery, offering clinically relevant information for patient management.

## Materials and methods

### Study design

**Fig. 1:**
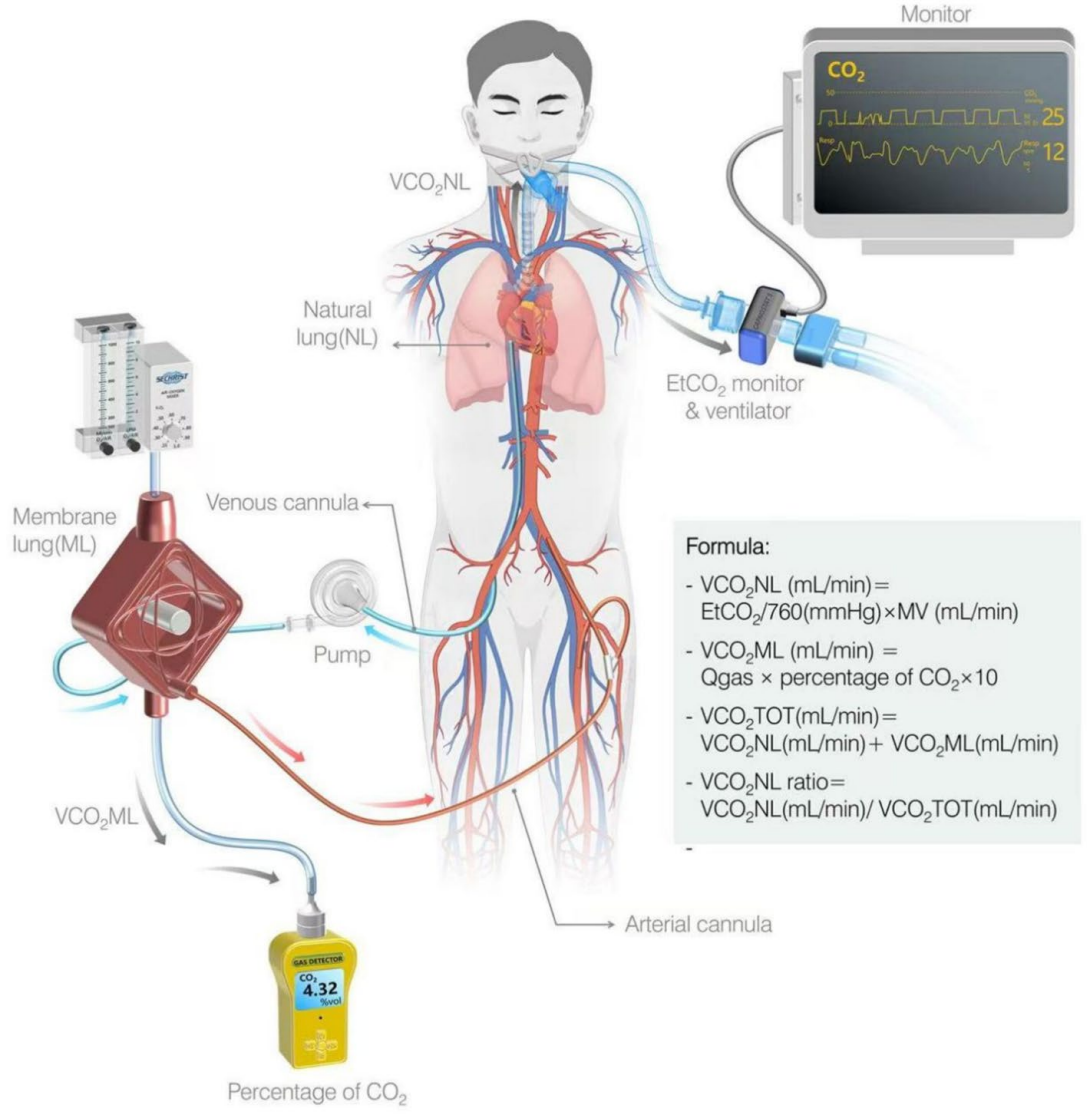
Study design. Note: the figure shows conceptual design only; the device appearances may vary from real products

This study is a prospective observational single centre study conducted in the Cardiac Intensive Care Centre, Beijing Anzhen Hospital, Capital Medical University. The entire study design is shown as illustrated (Fig. 1). Beijing Anzhen Hospital is a tertiary comprehensive hospital and a clinical teaching institution affiliated with Capital Medical University. The study protocol was approved by the Ethics Committee of Beijing Anzhen Hospital, Capital Medical University. The ethics approval number is 2020189X.

All consecutive patients admitted to our centre during the study period were screened for eligibility using predefined inclusion/exclusion criteria at the time of ECMO initiation. Inclusion criteria included: (1) patients older than 18 years; (2) patients with refractory CS requiring VA-ECMO. Exclusion criteria included: (1) patients with either pulmonary diseases or impaired oxygenation/CO_2_ clearance (PaO_2_/FiO_2_ < 200 mmHg or PaCO_2_ > 50 mmHg) throughout the entire study period; (2) patients not mechanically ventilated during the study period; (3) missing data. Patients were monitored daily after inclusion, with relevant study data collected. Those who met any exclusion criteria during follow-up were promptly withdrawn. Cardiogenic shock was defined when the following conditions were met: (1) systolic blood pressure < 80–90 mmHg despite adequate filling volumes, use of multiple adrenergic agents, or an IABP, (2) cardiac index < 2.2 L/min/m^2^, (3) metabolic acidosis (i.e., pH < 7.3 with serum lactate > 3.0 mmol/L), and (4) end-organ hypoperfusion (urine output < 30 mL/h) [13].

### ECMO management

The protocols for VA-ECMO initiation and management have been previously described [14]. In brief, peripheral VA-ECMO was established via femoral cannulation using a semi-open technique, with systematic placement of an additional 6 Fr distal perfusion catheter to prevent limb ischemia. All procedures were conducted by an experienced ECMO team. ECMO blood flow was titrated according to clinical parameters, including mixed venous oxygen saturation, perfusion status, lactate clearance, and mean arterial pressure (MAP). Systemic anticoagulation with unfractionated heparin targeted an activated clotting time (ACT) of 180–210 s or an activated partial thromboplastin time (aPTT) 1.5–2 times baseline. ECMO-related complications were closely monitored.

Patients underwent a standardized weaning trial upon meeting institutional criteria [7, 15], which included: (1) Hemodynamic stability (MAP > 60 mmHg) for ≥ 24 h with minimal/no vasopressor support, (2) Pulsatile arterial waveform, pulse pressure ≥ 15–20 mmHg (during IABP pause), (3) Baseline ECMO flow ≤ 1.5 L/min, (4) LVEF ≥ 20–25%, (5) Resolution of major metabolic derangements, lactate < 2.0 mmol/L, pH 7.35–7.45 and electrolyte balance. To evaluate CO_2_ parameters across the spectrum of cardiac recovery and control for inter-patient variability in ECMO flow, we implemented a rigorous daily weaning assessment protocol: (1) Safety-first approach: daily weaning trials were conducted by first reducing ECMO flow to 2 L/min for assessment and data collection; (2) Stratified assessment: hemodynamically stable patients proceeded to ≤ 1.5 L/min for formal weaning evaluation, while unstable patients were recorded as failures at 2 L/min and returned to baseline support.

### Ventilation settings

Mechanical ventilation was protocolized with tidal volumes of 6–8 mL/kg, PEEP 5–10 cmH_2_O, and FiO_2_ titrated to PaO_2_ > 60 mmHg. The membrane oxygenator’s sweep gas flow was adjusted based on ECMO blood flow (typically 1:1 to 1:2 ratio of gas-to-blood flow) to ensure adequate CO_2_ clearance and maintain normocapnia. Ventilator and ECMO settings were dynamically coordinated during weaning trials [16].

### Data collection

Baseline demographic, haemodynamic, laboratory, and follow‐up outcome data were collected via medical record review. Haemodynamic and laboratory parameters were recorded at baseline, prior to VA‐ECMO initiation, and before weaning trials. Ventilator settings and other respiratory parameters were recorded daily following VA‐ECMO insertion. The end-tidal carbon dioxide partial pressure (EtCO_2_) levels were measured using a capnograph (Netherlands, Philips) to calculate native lung CO₂ exhalation (VCO_2_NL formula 1). The capnograph was connected in-line within the ventilator circuit. CO₂ concentration at the membrane oxygenator outlet was measured using a CO₂ detector (USA, Ormond Beach) from which membrane lung CO₂ exhalation (VCO_2_ML, formula 2) was derived [17]. The CO₂ detector is tubing-linked to the oxygenator’s gas outlet. Total CO₂ removal (VCO_2_TOT formula 3) equals the sum of VCO_2_NL and VCO_2_ML. The ratio of VCO_2_NL to VCO_2_TOT (VCO_2_NL ratio formula 4) was used as a surrogate for the proportion of pulmonary artery blood flow to total systemic flow [17].

1 $$ {\text{VCO}}_{{2}} {\text{NL }}\left( {{\text{mL}}/{\text{min}}} \right) = {\text{EtCO}}_{{2}} /{76}0\left( {{\text{mmHg}}} \right) \times {\text{MV }}\left( {{\text{mL}}/{\text{min}}} \right) $$

2 $$ {\text{VCO}}_{{2}} {\text{ML }}\left( {{\text{mL}}/{\text{min}}} \right) = {\text{Qgas}} \times {\text{percentage of CO}}_{{2}} \times {1}0 $$

3 $$ \begin{aligned} {\text{VCO}}_{2} {\text{TOT }}\left( {{\text{mL}}/{\text{min}}} \right) & = {\text{VCO}}_{2} {\text{NL }}\left( {{\text{mL}}/{\text{min}}} \right) \\ & + {\text{VCO}}_{2} {\text{ML }}\left( {{\text{mL}}/{\text{min}}} \right) \\ \end{aligned} $$

4 $$ {\text{VCO}}_{{2}} {\text{NL ratio}} = {\text{VCO}}_{{2}} {\text{NL }}\left( {{\text{mL}}/{\text{min}}} \right)/{\text{VCO}}_{{2}} {\text{TOT }}\left( {{\text{mL}}/{\text{min}}} \right) $$

VCO_2_ML (mL/min): CO_2_ clearance volume of the membrane oxygenator, VCO_2_NL (mL/min): CO_2_ exhalation of the patient's natural lung, VCO_2_TOT (mL/min): total carbon dioxide removal, MV (mL/min): minute ventilation volume, Qgas (L/min): gas flow of the membrane oxygenator, percentage of CO_2_ (%): CO_2_ concentration of the membrane oxygenator gas outlet, EtCO_2_ (mmHg): end-tidal CO_2_ pressure.

### Study outcomes

The primary endpoint was the predictive performance of EtCO_2_ and VCO_2_NL ratio for successful VA-ECMO weaning. Successful weaning was defined as survival without ECMO re-initiation within 48 h after decannulation (regardless of LVAD or heart transplantation status), while weaning failure was defined as death or ECMO reinitiation within 48 h of decannulation.

### Statistical analysis

Continuous variables such as age, body mass index, mean arterial pressure, lactate, and pH were found to follow non-normal distributions. Categorical variables are expressed as count (percentage [%]) and continuous variables as median (interquartile range). Group differences were assessed using the Wilcoxon rank-sum test for continuous variables and Fisher’s exact test or chi-squared test for categorical variables. Multivariable logistic regression was used, with statistically significant (*p* < 0.10) and clinically relevant predictors included. The predictive performance of EtCO_2_ and VCO_2_NL ratio was evaluated using receiver operating characteristics (ROC) curves, with area under the curve (AUC) and 95% confidence intervals (CIs) calculated. Optimal thresholds were determined by minimising the explicit cost ratio, equivalent to maximising Youden’s index. Statistical significance was defined as *p* < 0.05. Finally, it should be noted that although multiple measurements per patient were included, we treated each weaning trial as an independent event for the purpose of predictive modeling, as each trial represented a distinct clinical decision point. We acknowledge the potential for clustering effects and have interpreted the results with this limitation in mind. All analyses were performed in the R environment (version 4.3.1, R Foundation).

## Results

###  Participants

**Fig. 2:**
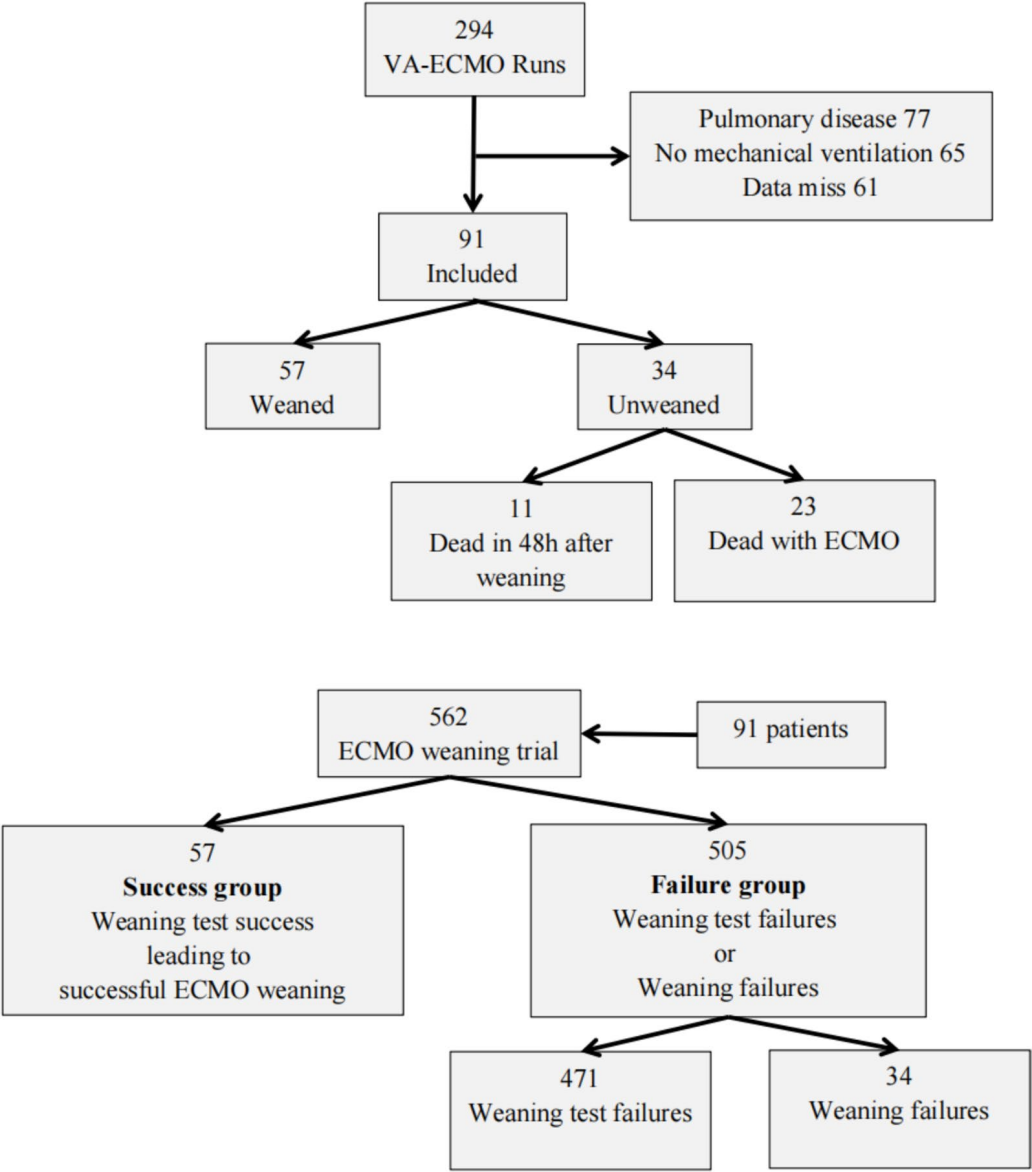
Study flow

A total of 294 adults who received VA-ECMO for refractory CS between January 2023 and December 2024 were screened. Of these, 91 patients were included in this analysis, resulting in 562 data points. These data points were analysed and stratified into the weaning test success leading to successful weaning group (abbreviated as the success group, N = 57) and the weaning test failures or weaning failures group (abbreviated as the failure group, N = 505) (Fig. 2). Baseline clinical characteristics are presented in Table 1. The median age of the study population was 62 [46, 65] years, and 77% of the patients were male. Most patients (71%) had unstable circulation following cardiac surgery. Baseline haemodynamic data indicators prior to ECMO were markedly low, and the dosage of vasoactive agents was relatively high. The median duration of ECMO support was 5 [4, 9] days, and the median hospital stay was 18 [11, 25 ] days. There were 57 patients (63%) successfully weaned and survived. The remaining 34 patients who failed to wean ultimately died. Of the 34 mortality cases, 23 deaths occurred under ECMO support secondary to unrecoverable low cardiac output syndrome, with the other 11 cases experiencing conservative treatment-resistant cardiac function exacerbation within 48 h post-decannulation.

**Table 1:** Baseline clinical characteristics

n = 91	
Age, years	59 [50, 66]
Male, %	70 (77)
Body mass index, kg/m^2^	25 [23, 26]
*Reason for VA-ECMO*	
Post cardiotomy, %	65 (71)
Acute myocardial infarction, %	20 (22)
Fulminant myocarditis, %	3 (3)
ECPR, %	3 (3)
*SCAI classification*	
C, %	10 (11)
D, %	68 (75)
E, %	13 (14)
*Clinical and biological variables before VA-ECMO implantation*	
PH	7.4 [7.32, 7.46]
Serum lactate, mmol/L	5 [3, 11]
CRRT,%	14 (15)
IABP, %	59 (65)
SAP, mmHg	60 [57,66]
DAP, mmHg	41 [32,45]
Pulse pressure, mmHg	15 [12,20]
SOFA score	9 [8, 11]
VIS, µg/kg/min	21 [12,31]
*Clinical outcome*	
Successful weaning, %	57 (63)
In-hospital death, %	46 (51)
Length of ECMO running, days	5 [4, 9]
Length of stay in hospital, days	18 [11, 25]

Data are presented as medians (25th–75th percentile) or n (%)

ECPR, extracorporeal cardiopulmonary resuscitation; CRRT, continuous renal replacement therapy; IABP, intra-aortic balloon pump; SAP and DAP, systolic and diastolic arterial blood pressure; SOFA score, the sequential organ failure assessment score; VIS, vasoactive-inotropic score, in μg/kg/min, was calculated as follows: dopamine + dobutamine + 100 × epinephrine + 100 × norepinephrine + 15 × milrinone

### CO_2_ measurement

**Fig. 3:**
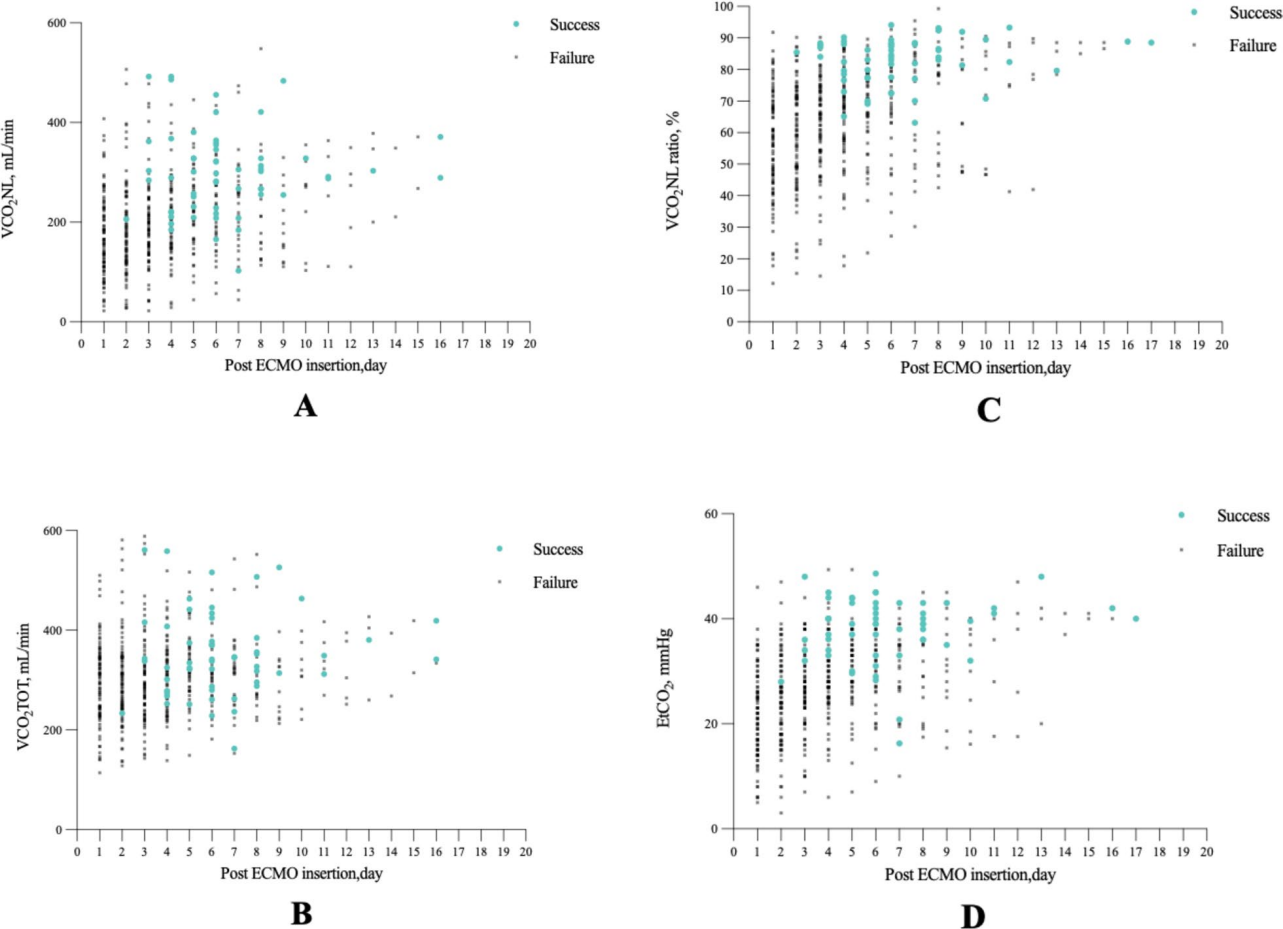
Plot of **A** VCO_2_NL (mL/min), **B** VCO_2_TOT (mL/min), **C** VCO_2_NL ratio (%), and **D** EtCO_2_ (mmHg) trends for 91 consecutive patients supported with VA-ECMO. Success = value on the day of successful weaning, Failure = value at all other timepoints (weaning test failures + weaning failures)

Respiratory data were collected daily following VA‐ECMO initiation. The trend for VCO_2_NL, VCO_2_TOT, the VCO_2_NL ratio, and end-tidal carbon dioxide partial pressure (EtCO_2_) for each patient is illustrated in Fig. 3. Respiratory and haemodynamic variables during the ECMO period, categorised by weaning test outcome, are presented in Table 2.

**Table 2:** Haemodynamic and respiratory data according to weaning outcome

n	Failure	Success	*p*
	505	57	
*Clinical and biological variables*			
Serum lactate, mmol/L	4 [2.5, 9.7]	1.6 [1.3, 2.4]	< 0.001
PH	7.43 [7.37, 7.49]	7.44 [7.42, 7.48]	0.194
SOFA score	8 [6, 9]	7 [5, 8]	0.001
VIS, ug/kg/min	13 [5.8,31.5]	8[5.5,13.3]	0.065
*Hemodynamic data*			
SAP, mmHg	104 [92,118]	115 [101, 124]	0.100
DAP, mmHg	58 [53, 68]	60 [56, 68]	0.992
Heart rate, beats/min	91 [74, 105]	89 [78, 100]	0.881
Pulse pressure, mmHg	25 [22, 58]	49 [40, 61]	0.013
Central venous pressure, mmHg	10 [7,12]	8[7,10]	0.320
LVEF	28 [15, 46]	39 [32, 50]	< 0.001
*Respiratory data*			
Respiratory rate, breaths/min	12 [12, 14]	12 [11, 14]	0.876
Tidal volume, mL/min	447 [375, 538]	480 [420, 559]	0.059
Minute ventilation, mL/min	5500 [4500, 6468]	5700 [5200, 6720]	0.084
Sweep gas flow, L/min	2 [2, 2.5]	1.5 [1, 2]	0.073
CO_2_%	4.5 [3.6, 5.7]	3.6 [2.8, 4.4]	< 0.001
VCO_2_ML, mL/min	99 [70, 131]	54 [40, 69]	< 0.001
VCO_2_NL, mL/min	195 [139, 260]	298 [231, 346]	< 0.001
VCO_2_TOT, mL/min	309 [250, 347]	342 [296, 408]	0.130
EtCO_2_, mmHg	27 [21, 34]	38 [35, 41]	< 0.001
VCO_2_NL ratio,%	67 [51, 77]	84 [79, 88]	< 0.001

Data are presented as medians (25th–75th percentile) or n (%)

SOFA score, the sequential organ failure assessment score; VIS, vasoactive-inotropic score, in μg/kg/min, was calculated as follows: dopamine + dobutamine + 100 × epinephrine + 100 × norepinephrine + 15 × milrinone

SAP and DAP, systolic and diastolic arterial blood pressure; LVEF, left ventricular ejection fraction; CO_2_%, CO_2_ concentration of the membrane oxygenator gas outlet

### Predictors of successful liberation from VA-ECMO

Respiratory and haemodynamic variables were recorded when ECMO flow was reduced to 2 L/min (Table 2). Serum lactate (4 [2.5,9.7] vs 1.6 [1.3, 2.4] mmol/L, *p* < 0.001) and SOFA score (8 [6, 9] vs 7 [5, 8], *p* = 0.001) were significantly higher in the failure group, while pulse pressure (25 [22, 58] vs 49 [40, 61], *p* = 0.013), LVEF (28 [15, 46] vs 39 [32, 50], *p* < 0.001), EtCO_2_ (27 [21, 34] vs 38 [35, 41], *p* < 0.001) and VCO_2_NL ratio ( 67 [51, 77] vs 84 [79, 88]) were significantly higher in the success group. EtCO_2_, the VCO_2_NL ratio, and pulse pressure were entered into two separate models. After adjustment for LVEF and SOFA score, both EtCO_2_ (odds ratio [OR] = 1.26, 95% confidence interval [CI] 1.17–1.37, *p* < 0.0001) and the VCO_2_NL ratio (1.14, 95% CI 1.09–1.21, *p* < 0.0001) remained independently associated with successful liberation from VA-ECMO (Table 3).

**Table 3:** Predictors of successful liberation from ECMO using multivariable logistic regression

Clinical variables	OR (95% CI)	*p*
*Model1*		
EtCO_2_	1.26 (1.17–1.37)	< 0.0001
LVEF	1.10 (1.05–1.16)	< 0.0001
Pulse pressure	1.01 (0.98–1.04)	0.4886
SOFA score	0.92 (0.76–1.10)	0.3652
*Model2*		
VCO_2_NL ratio	1.14 (1.09–1.21)	< 0.0001
LVEF	1.10 (1.04–1.16)	0.0005
Pulse pressure	1.01 (0.99–1.04)	0.3513
SOFA score	0.87 (0.73–1.03)	0.105

### Accuracy of EtCO_2_ and the VCO_2_NL ratio in predicting weaning success

**Fig. 4:**
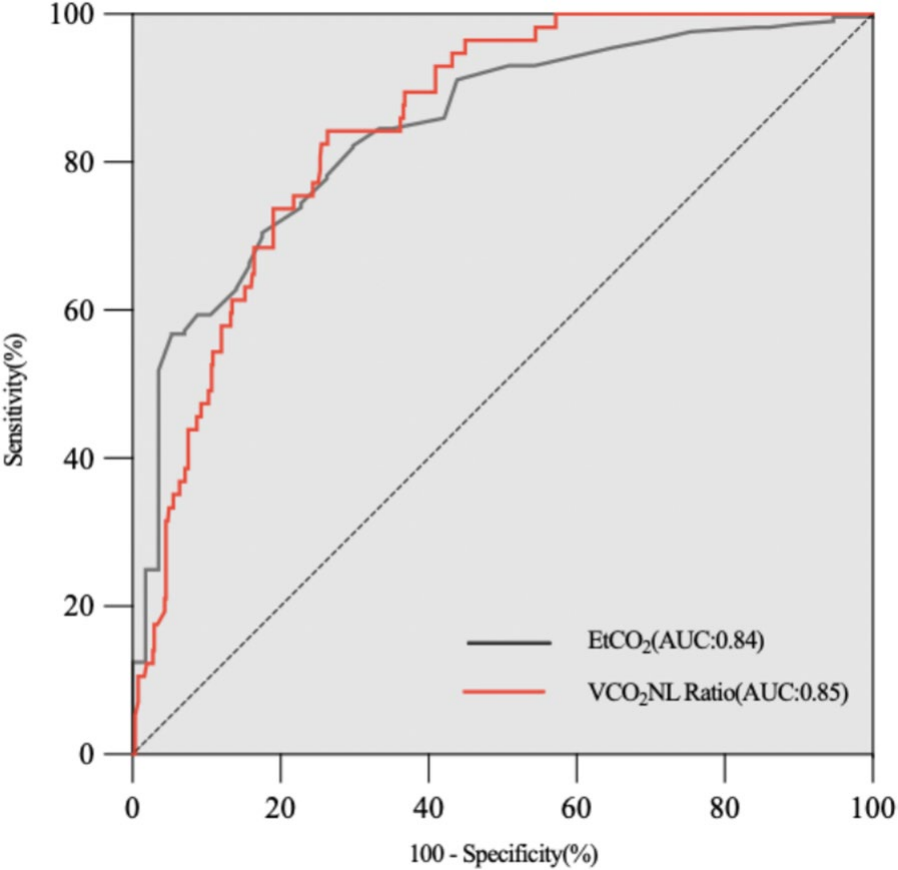
ROC curves of VCO_2_NL Ratio and EtCO_2_ for predicting weaning outcome. AUC and 95% CI were calculated based on 562 data points

Receiver operating characteristics (ROC) curves are shown in Fig. 4. The optimal cut-off values for predicting successful weaning were 34 mmHg for EtCO_2_ (sensitivity = 74.46%, specificity = 77.19%, AUC = 0.84), 79% for VCO_2_NL Ratio (sensitivity = 75.44%, specificity = 78.22%, AUC = 0.85) (Table 4).

**Table 4:** ROC AUCs of VCO_2_NL Ratio and EtCO_2_ for predicting weaning outcome

Clinical variables	Optimal cutoff	Sensitivity	Specificity	AUC
EtCO_2_, mmHg	34	74.46	77.19	0.84
VCO_2_NL Ratio,%	79	75.44	78.22	0.85

## Discussion

This prospective study demonstrates that EtCO_2_ and the VCO_2_NL ratio which is the ratio of VCO_2_NL to VCO_2_TOT serve as reliable indicators for predicting successful VA-ECMO weaning in patients with severe CS. Both parameters showed significant correlations with weaning success, with optimal predictive thresholds of EtCO_2_ > 34 mmHg and VCO_2_NL ratio > 79%. The findings suggest that routine monitoring of EtCO_2_ and the VCO_2_NL ratio may facilitate timely weaning decisions, thereby reducing risks associated with prolonged VA-ECMO support, including bleeding, thrombosis, and infection. As adjunctive tools to standard echocardiographic evaluation, these non-invasive real-time parameters provide a novel insight for ECMO weaning decision-making. Their integration with established markers may refine weaning protocols and ultimately enhance clinical outcomes.

In the initial management of severe CS, VA-ECMO plays a critical role in restoring systemic perfusion, thereby preventing multi organ failure and improving survival [18, 19]. However, prolonged VA-ECMO support in clinically stable patients carries significant risks, including haemorrhage, thrombosis, thrombocytopenia, limb ischaemia, vascular injury, infection, and organ dysfunction [20]. Thus, timely weaning is essential once cardiac function recovers. Currently, determining the optimal weaning timing remains a clinical challenge. Successful weaning primarily depends on the degree of cardiac recovery, typically assessed through haemodynamic parameters, echocardiographic indices, and pulmonary artery catheter data. Recent studies have explored potential predictors of successful VA-ECMO liberation. For example, Bo Ram Lee et al. demonstrated that pulse pressure (PP) during the first 12 h of VA-ECMO support, particularly when adjusted for vasoactive-inotropic score (VIS), correlates with cardiac recovery and predicts successful weaning [21]. Echocardiographic markers, such as improvements in lateral e' velocity and tricuspid annular S' velocity, have also been associated with cardiac function [4]. Consistent with these findings, our observations confirm that successful weaning correlates with higher PP, stable heart rate, and favourable echocardiographic parameters. However, existing predictors have notable limitations. Haemodynamic measures such as PP can be affected by left ventricular venting, aortic valve insufficiency, or intra-aortic balloon pump use. While echocardiography provides valuable structural and functional insights, it does not offer continuous, real-time monitoring. Thus, a multimodal approach, combining dynamic haemodynamic assessment, serial echocardiography, and clinical evaluation, is essential for optimising weaning decisions and improving outcomes in patients with CS.

In recent years, EtCO_2_, the partial pressure of CO_2_ in alveolar gas at the end of expiration, has gained attention as a potential haemodynamic marker. Since EtCO_2_ levels depend on pulmonary artery blood flow, venous return enables CO_2_ transport to the lungs, where it is removed via gas exchange. A decline in EtCO₂ (assuming stable CO_2_ production and alveolar function) reflects reduced venous return and pulmonary perfusion, increasing alveolar dead space [11, 22, 23]. Prior research has demonstrated a strong correlation between CO_2_ elimination and pulmonary artery blood flow, which closely reflects cardiac output [10]. Recent studies further support this association, particularly in VA-ECMO patients requiring left ventricular venting [24–26]. In our study, EtCO_2_ showed a strong association with successful weaning, serving as a proxy for myocardial reserve. A threshold of > 33.95 mmHg yielded high predictive value. To minimise confounding factors (e.g., hyperventilation, hypoventilation, fever), we introduced the VCO_2_NL ratio (see Formula 1–4), which represents the proportion of CO_2_ produced by the native lungs relative to total CO_2_ output. Given the short-term stability of oxygen consumption, the total CO_2_ production remains constant; thus, variations in this ratio reflect changes in pulmonary artery blood flow, and by extension, cardiac function. Notably, the VCO_2_NL ratio outperformed EtCO_2_ alone in predicting successful weaning. Based on these findings and previous studies, improvements in EtCO_2_ and VCO_2_NL ratio during VA‐ECMO maintenance may indicate early cardiac recovery and help guide clinical interventions. EtCO_2_ and the VCO_2_NL ratio can be monitored non-invasively via infrared capnography in mechanically ventilated patients. A simple variable (EtCO_2_/VCO_2_NL ratio) could serve to assess weanability while the patient is still on ECMO, or to stop the weaning trial early and return the patient to baseline ECMO to avert additional cardiovascular stress. Future integration of these parameters with other haemodynamic markers could further improve weaning prediction accuracy.

Our research has several limitations. First, as a single-centre retrospective study with a modest sample size and tightly controlled ventilation parameters, generalisability may be limited. The ECMO weaning predictors we identified require prospective validation in broader patient populations across multiple institutions. Second, the non-randomised design of the registry introduces the possibility of unmeasured confounding variables. For example, physician-led decisions regarding systolic blood pressure targets, PP thresholds, and vasopressor usage during VA-ECMO support were made without standardised protocols. Third, some weaning trials were conducted in patients with minor metabolic derangements or low native cardiac output. While this may introduce heterogeneity, it reflects real-world scenarios and strengthens the generalizability of our thresholds. Fourth, the absence of systematic daily echocardiographic assessments (particularly LVOT TVI and TDSa measurements) represents a significant constraint. While these parameters’ routine measurement was hindered by the frequent suboptimal acoustic windows in post-cardiotomy patients, reflecting real-world clinical challenges. Future multicenter studies should implement standardized echocardiographic protocols to validate whether CO_2_ metrics provide additive value beyond traditional echocardiographic assessments. Fifth, the absence of invasive cardiac output monitoring, such as pulmonary artery catheterisation, the gold standard, limits our ability to definitively characterise the relationship between EtCO_2_ and cardiac output. Future investigations should incorporate comprehensive haemodynamic monitoring and employ multicentre prospective designs to confirm our findings.

## Conclusion

This study suggests that the VCO_2_NL ratio and EtCO_2_, readily measurable and computable parameters, may serve as practical predictors of successful VA-ECMO weaning. Thresholds of EtCO_2_ > 34 mmHg and VCO_2_NL ratio > 79% predicted successful weaning with high accuracy. Further research is needed to determine whether the integration of these indicators can inform clinical decision-making, reduce complications associated with VA-ECMO, and improve patient prognosis.

## Supplementary Information

Supplementary material 1.

## Data Availability

The datasets used and analyzed during the current study are available from the corresponding author on reasonable request.

## Abbreviations

VA-ECMO: Venoarterial extracorporeal membrane oxygenation
CS: Cardiogenic shock
CO_2_: Carbon dioxide
EtCO_2_: End-tidal carbon dioxide
VCO_2_NL: Native lung carbon dioxide elimination
VCO_2_TOT: Total carbon dioxide elimination
PAC: Pulmonary artery catheter
PICCO: Pulse indicator continuous cardiac output
PP: Pulse pressure
AUC: Area under the curve
ROC: Receiver operating characteristics
CSI: Confidence interval
VIS: Vasoactive-inotropic score
