# Supplementary figures and images for: Carbon dioxide elimination as a guide to venoarterial extracorporeal membrane oxygenation weaning: a prospective observational study
Source: Ann Intensive Care. 2025 Oct 14;15:157. doi: 10.1186/s13613-025-01583-4 (PMC12521700; doi:10.1186/s13613-025-01583-4)

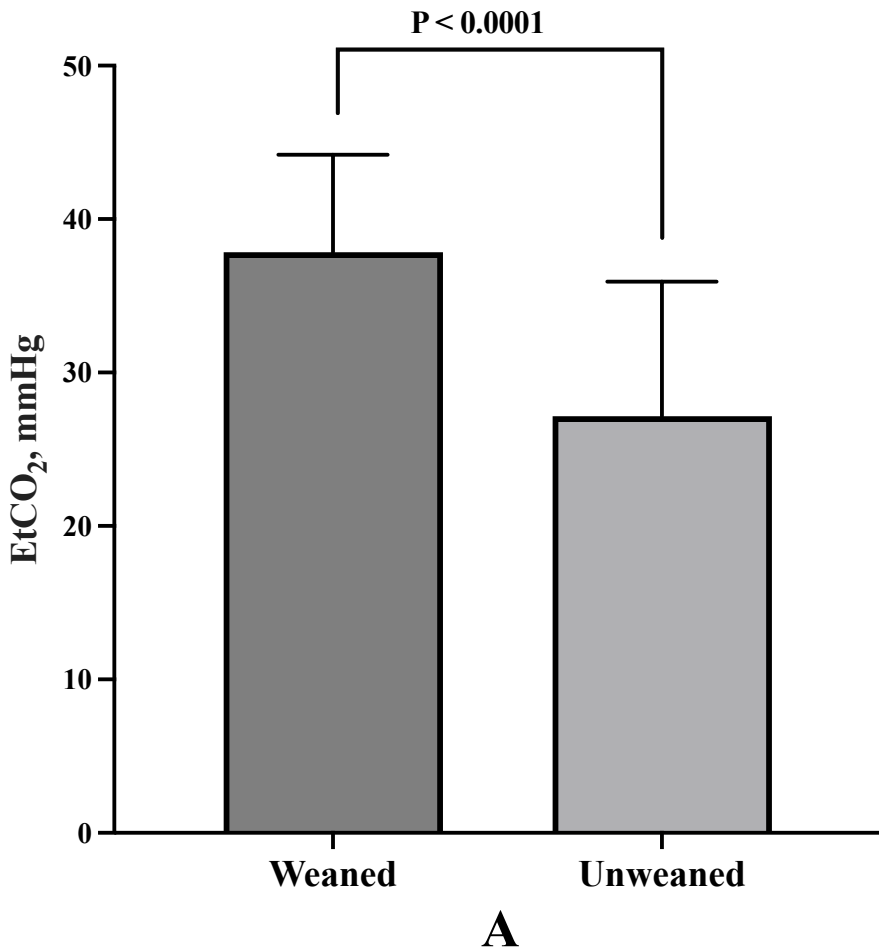

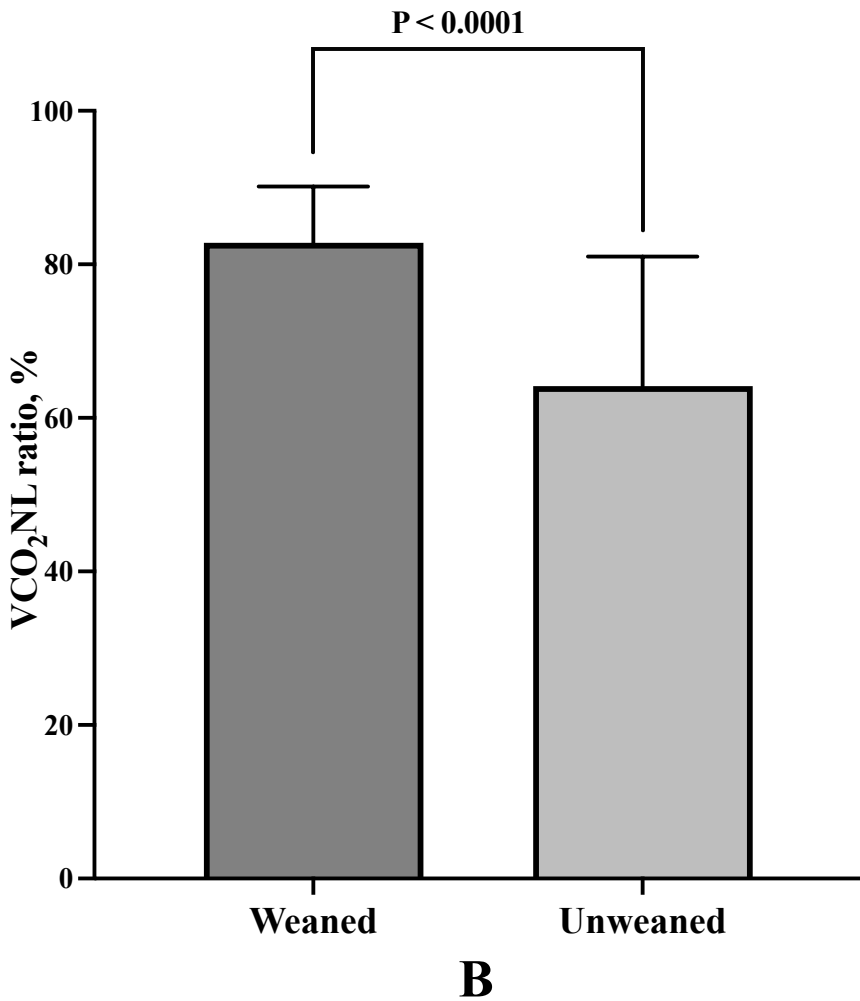

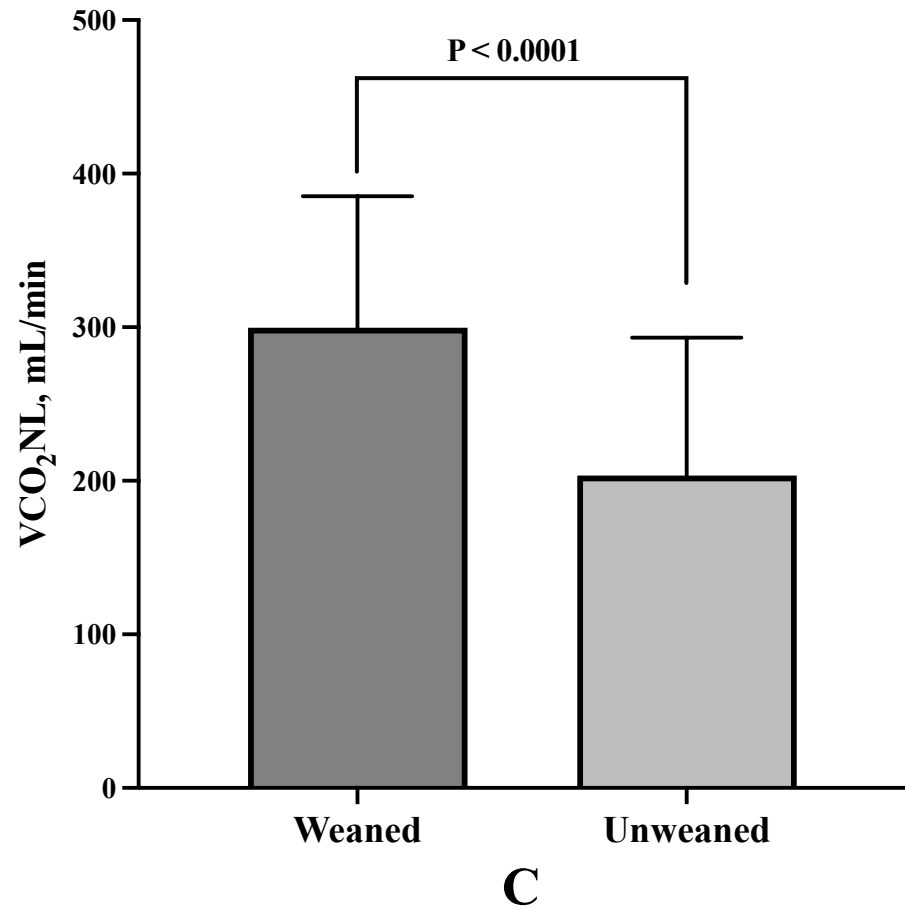

**P = 0.130**

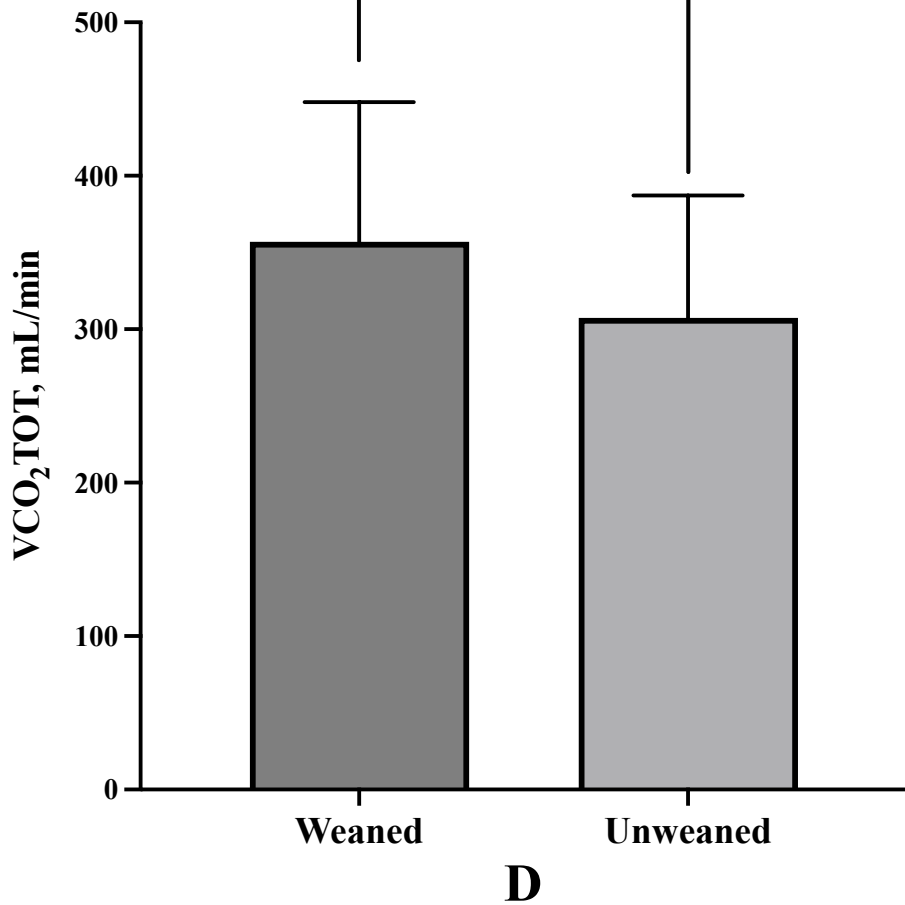

Supplement: Supplementary file 1 — Supplementary material 1. [file 13613_2025_1583_MOESM1_ESM.pdf]
